# Kappa and Mu Opioid Receptors in Chronic Cough: Current Evidence and Future Treatment

**DOI:** 10.1007/s00408-025-00812-8

**Published:** 2025-05-13

**Authors:** Surinder S. Birring, Peter V. Dicpinigaitis, Toby M. Maher, Stuart B. Mazzone, Clive P. Page, Amale Hawi, Thomas Sciascia, Alyn H. Morice

**Affiliations:** 1 Centre for Human & Applied Physiological Sciences, School of Basic & Medical Biosciences, King’s College London, London, UK; 2 Albert Einstein College of Medicine and Montefiore Medical Center, Bronx, NY USA; 3 University of Southern California, Los Angeles, CA USA; 4 Fibrosis Research Group, National Heart and Lung Institute, Imperial College London, London, UK; 5 University of Melbourne, Melbourne, VIC Australia; 6 Trevi Therapeutics, Inc, New Haven, CT USA; 7 Respiratory Medicine, Hull York Medical School, Castle Hill Hospital, Castle Rd, E Yorkshire, Cottingham, HU16 5JQ UK

**Keywords:** Chronic cough, Hypersensitivity, Kappa agonism, Mu antagonism, Nalbuphine, Opioid receptors

## Abstract

Chronic cough is a significant burden on patient quality of life and is associated with poor health outcomes. Chronic cough may be a result of neural hypersensitivity due to changes in both the peripheral and the central nervous systems, although the exact mechanisms underlying its pathogenesis are not completely understood. Opioid receptors, specifically kappa and mu, are potential therapeutic targets in the management of chronic cough because they play a pivotal role in both the peripheral and the central neural pathways implicated in the act of coughing. Morphine, a mu opioid receptor agonist, is an effective cough modulator; however, mu receptor agonists are part of a drug class that can induce respiratory depression and euphoria, with strong reinforcing properties that may lead to excessive use and abuse. Drugs with a dual-acting mechanism of kappa receptor agonism and mu receptor antagonism may be effective in the management of chronic cough without the potential for abuse. This review summarizes the current understanding of the mechanisms of cough hypersensitivity, the role of the kappa and mu receptors in the neurophysiology of cough, and the clinical potential of targeting these receptors as a novel way of managing chronic cough.

## Introduction

Chronic cough in adults is defined as a cough that lasts for 8 weeks or more [1, 2]. The prevalence of chronic cough is approximately 5–10% globally [3–5]. Chronic cough is most common among older patients (aged > 50 years) and is more prevalent in women than in men in the vast majority of studies [6, 7]. It is a comorbid condition that occurs in tandem with respiratory conditions, such as asthma, chronic obstructive pulmonary disease (COPD), rhinosinusitis, idiopathic pulmonary fibrosis (IPF), and other interstitial lung diseases (ILDs) [8–11]. Refractory chronic cough (RCC) is a cough that persists despite guideline-based treatment for the underlying disease, and unexplained chronic cough (UCC) is a chronic cough for which no cough-associated conditions are diagnosed [1]. Chronic cough may be a result of neural hypersensitivity [12] and, although the exact mechanisms underlying its pathogenesis are not completely understood [4], it is suggested that changes in both the peripheral nervous system (PNS) and the central nervous system (CNS) are involved [12].

The substantial burden of chronic cough has a significant impact on work productivity, interpersonal relationships, and healthcare resources [4], and, because of this, patients commonly consult with primary care physicians [9]. The impact of chronic cough on daily quality of life is considerable, and the impact is estimated to be similar to that of respiratory lung disease, such as asthma, bronchiectasis, and COPD [13]. In IPF specifically, chronic cough is associated with disease progression and poor health outcomes [11, 14, 15].

Many treatments are used to manage chronic cough, such as neuromodulators, including low-dose morphine, gabapentin, and pregabalin, or non-pharmacological approaches, such as speech therapy or physiotherapy [1]. However, chronic cough is often refractory to treatment and there is a need for better therapies. Recently, the P2X3 antagonist gefapixant was approved for use in managing RCC and UCC in many countries, including those in the European Union, Switzerland, United Kingdom, and Japan; it is not approved in the United States [16–19]. We summarize the current understanding of the mechanisms of cough hypersensitivity and the potential of novel dual-acting agonist–antagonist opioid receptor-targeting drugs for management of chronic cough.

## Overview of the Mechanisms of Chronic Cough and Cough Hypersensitivity

Cough is characterized by a forced expulsive maneuver, usually against a closed glottis, and is accompanied by a characteristic sound [20]. Under normal conditions, cough provides a crucial protective function of the lungs, preventing aspiration [21]. Cough involves a neural pathway in which sensory nerves in the airways are activated, leading to an alteration in the respiratory pattern via complex circuitry in the CNS. Three types of cough have been classified on the basis of their central control mechanisms: type I (reflex cough), type II (voluntary cough), and type III (evoked cough) [22].

The primary distinction between evoked and reflex cough depends on the intensity of the stimulus [22]. Coughing typically happens when an irritating stimulus is detected, triggering a sensation known as the urge to cough [23]. This sensation involves the activation of several brain regions: the primary sensory and motor cortex, the insula, the orbitofrontal cortex, discrete areas of the anterior cingulate cortex, and the cerebellum [23]. Weak stimuli (such as strong odors, e.g., perfume, or a tickling sensation in the throat) lead to an urge to cough sensation followed by an evoked cough, while strong stimuli induce reflex cough via brainstem circuitry [22]. Evoked cough engages medullary and supramedullary CNS structures in a mechanistic interplay [22]. Often, patients can consciously suppress evoked cough [21, 22]; however, reflex cough becomes inevitable as the intensity of the stimulus increases [22]. Reflex cough engages the afferent nerves in the pulmonary tissue, sending signals to the sensory and respiratory nuclei of the brainstem. These afferent nerve fiber inputs result in the activation of the efferent nerve fibers responsible for the coordinated neuromuscular act of coughing through their innervation of thoracic structures [4, 21, 22]. Examples of reflex cough are aspiration of a foreign body into the airway or experimentally induced cough with high concentrations of inhaled tussive agents such as capsaicin [22]. In contrast with reflex cough, voluntary cough involves supramedullary CNS structures and is initiated consciously, independent of external stimuli [22]. Functional magnetic resonance imaging in patients with evoked or voluntary cough shows activation in multiple areas, expressing opioid receptors such as the kappa opioid receptors (KORs) and the mu opioid receptors (MORs), within the mid-cingulate cortex, insula, amygdala, basal ganglia, thalamus, and brainstem [22, 24].

Chronic cough occurs in patients who have pulmonary conditions that affect the airway, such as IPF, asthma, and rhinosinusitis, and other conditions such as extra-esophageal reflux disease or airway reflux [9–11, 21, 25]. It is suggested in the evolving hypothesis that local tissue inflammation-induced effects contribute to chronic cough and that chronic cough is associated with altered neurophysiology [9, 12]. The disparate chronic cough-related patient populations are collectively recognized under the clinical concept of cough hypersensitivity syndrome (CHS) [9]. The sensorimotor phenomenology of cough suggests that CHS is attributed to increased sensitivity of the peripheral vagus nerve or an imbalance of excitatory/inhibitory function of the CNS [4, 9].

## Neurophysiology in Chronic Cough

The neurophysiological concept of sensitization may explain the development of chronic cough. *Sensitization* refers to the phenomena of increased responsiveness of peripheral and central nociceptive neural pathways to their normal or subthreshold afferent input [9, 26]. Therefore, the concept of sensitization could provide insight into CHS, in which coughing is more readily activated with subnormal sensitivity thresholds [4]. The idea of sensitization encompasses multiple neurophysiological mechanisms that separately or collectively may be relevant to addressing the underlying cause of chronic cough. Understanding these mechanisms supports the development of drugs designed to target opioid receptors, which are densely populated in the regions of the CNS and PNS that regulate sensitization [22, 24]. Much of what is understood about the development of sensory hypersensitivity states comes from studies of chronic pain and chronic itch in which common mechanisms have been identified: namely, peripheral sensitization, wind-up, and central sensitization [27, 28].

Peripheral sensitization commonly occurs in afferent sensory receptors under conditions of local tissue damage that alters the biology of afferent nerve ending–local tissue homeostasis [29]. These peripheral nerves are responsible for the transmission of information arising from tissue damage to the CNS and include A-delta fibers and C-fibers [30, 31], which, among other receptors, express the KORs and MORs on their nerve endings [32, 33]. In peripheral sensitization, the afferent nerve fibers become more excitable, making other triggering stimuli more likely to cause afferent nerve activation [34], thereby increasing the sensory drive to evoke coughing.

In contrast, the process of central sensitization involves mechanisms in the CNS that amplify sensory nerve fiber inputs, such that even normal afferent nerve fiber activity can produce heightened cough responses [9, 27]. The mechanisms leading to central sensitization have been studied extensively in spinal pain circuits, where it has been suggested that it is promoted through the activation of neurokinin receptors, leading to a gradual depolarization that alleviates the magnesium ion (Mg^2+^) block on *N*-methyl-d-aspartate receptors expressed by spinal dorsal horn neurons [27]. In addition, dorsal horn inflammation is believed to be important in maintaining centrally sensitized states, through a process orchestrated by spinal glial cells [27]. The evidence supporting a role for central sensitization in chronic cough is growing [9]. Notably, and consistent with prior chronic pain studies in human and animal models, patients with chronic cough show central sensitization effects associated with reduced excitability thresholds in brain structures that extend from the upper brainstem to the cortex [9, 27].

## The Role of KOR and MOR in Chronic Cough

Opioid receptors are a family of homologous cell surface, G-protein-coupled receptors [31, 35] and are expressed throughout the CNS and the PNS [35]. The three most well-studied opioid receptors are KOR, MOR, and delta opioid receptor (DOR) [35]. Opioid receptors trigger activation of adenylate cyclase, leading to an increase in cyclic adenosine monophosphate (cAMP) production and, thus, elicit stimulatory or inhibitory actions. The elevated cAMP levels can activate protein kinase A, which causes phosphorylation of various proteins, ion channels, and enzymes, subsequently leading to their activation or inhibition [36]. There are more than 20 endogenous opioids, each with unique selectivity and signaling across the different opioid receptors [37]. Primarily, dynorphins, endorphins, and enkephalins are the endogenous opioids for KOR, MOR, and DOR, respectively [38]. However, the endogenous opioids do not exhibit exclusive activity via the respective opioid receptor [38]. The diversity of the opioid receptors and endogenous opioids may exist to facilitate the precise fine-tuning of physiological responses [37].

### Opioid Receptor Expression

**Fig. 1:**
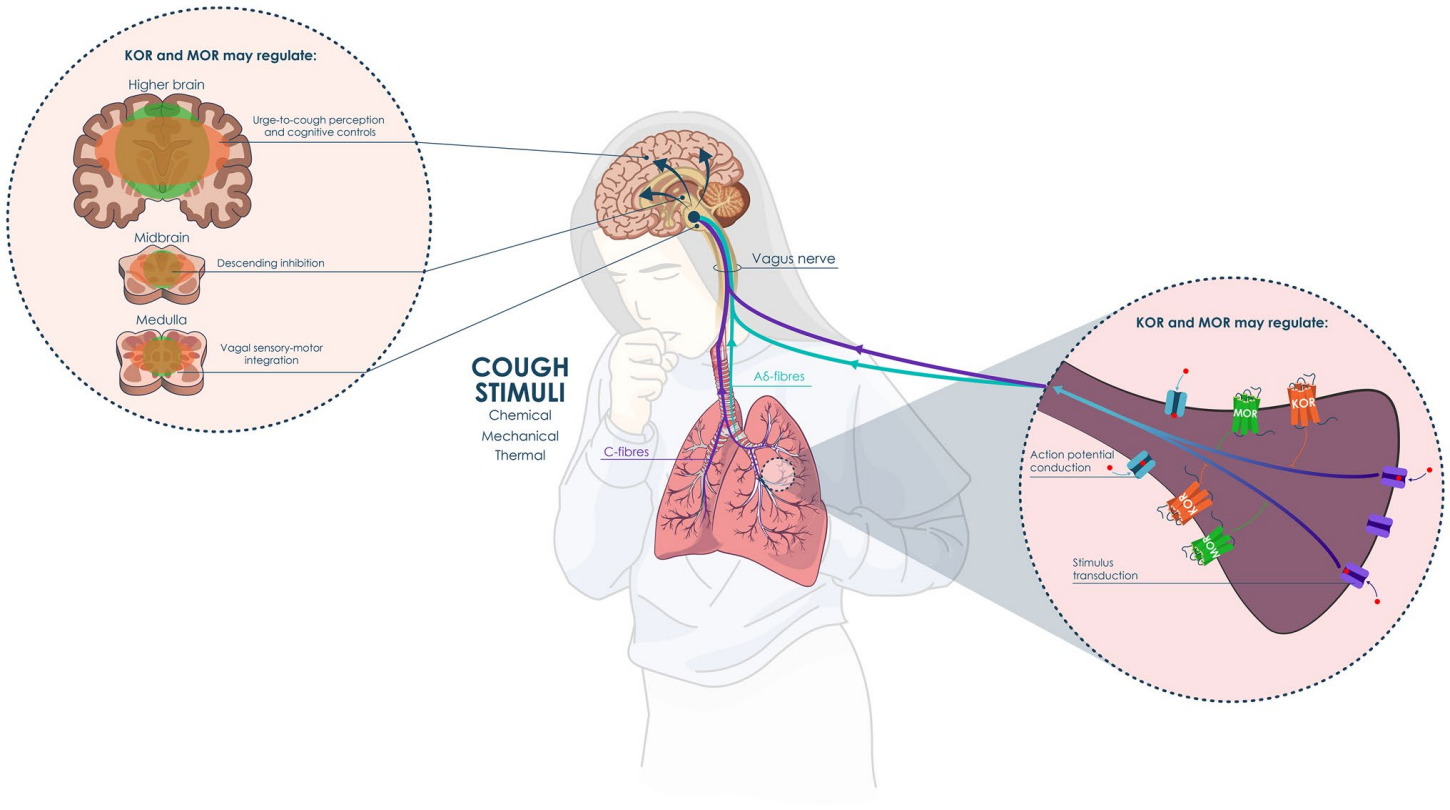
The peripheral and central processes contributing to cough

The KORs and MORs are crucial to regulating affective states, neuroendocrine and autonomic stress responses, and mood and motivational states [39]. These receptors are widely expressed throughout the CNS, the respiratory system, and the PNS [24, 39]. Peripherally, the most abundant sites of opioid receptor expression are within the structures central to the regulation of bronchial and pulmonary vascular responses, including the vagus nerve [40, 41]. Opioid receptors are also located in the pulmonary neuroendocrine cells and sensory C-fibers within the bronchial epithelium [33] and in the peripheral endings of A-delta fibers and C-fibers, which innervate the thoracic structures [32–34]. Centrally, MORs are highly expressed in the anterior cingulate cortex, insula, amygdala, brainstem, and spinal cord [32]. With respect to the respiratory system, these structures all contribute to the control of pulmonary ventilation mechanics, respiratory reflexes, and the perception of respiratory sensations [9]. A high density of KORs exists in the frontal cortex, insula, and amygdala [42]. Multiple endogenous opioids with different relative affinities for opioid receptor subtypes are found in the medullary and pontine respiratory regions, and all three classic opioid receptors are found in the respiratory-related regions of the brainstem and spinal cord [43] (Fig. 1).

## KORs and MORs as Therapeutic Targets in Chronic Cough

The neurobiological pathways responsible for hypersensitivity in chronic cough, pain, and itch overlap [28, 44]. Given the role and expression of KORs and MORs in the CNS and the PNS, it is not surprising that results of preclinical studies have shown that KOR and MOR agonists and antagonists can modulate the sensitivity of peripheral vagal afferent nerves [45–47]. This influence may extend to other peripheral processes, such as regulation of cholinergic neurotransmission in the airway, ultimately impacting airway tone [46]; thus, the rationale for investigating opioid receptor-directed pharmacological interventions is clear. This drug class has the potential to modulate both the threshold set point in the transition from evoked cough to reflex cough and the pathological effects of central sensitization. Results from preclinical studies have shown that KORs and MORs mediate the anti-tussive effects of codeine, supporting their role as important therapeutic targets for chronic cough [48–50]. Other study results have shown that KORs are involved in the modulation of inflammation [51, 52], including inflammatory processes in the lungs [53], possibly by reducing the inflammatory response of alveolar macrophages [54].

Morphine is a MOR agonist that also exhibits partial agonistic activity on KORs [55–57]. MOR agonists belong to a class of drug known to induce respiratory depression and euphoria and to possess strong reinforcing properties, potentially leading to excessive use and abuse [39, 58]. Administering MOR agonists and mixed MOR/KOR and MOR/DOR agonists in animal models increased extracellular dopamine levels in the shell of the nucleus accumbens; it is suggested that this increase contributes to the development of the reinforcing properties of many drugs of abuse in humans [59–62]. Low-dose morphine is used in the management of chronic cough and is recommended in current guidelines [1, 57]. Because there is a lack of a dose–response relationship with morphine as an anti-tussive agent, higher doses of morphine such as those used in pain management are not recommended for the management of chronic cough [1].

Treatment with slow-release morphine (5 mg or 10 mg twice daily) is well tolerated, with minimal sedation effects in patients with refractory chronic cough [57]. In patients who responded to low-dose morphine sulfate, cough frequency was reduced by 71.8% compared with placebo over a 24-h period [56]. In another study [63], patients with chronic cough due to IPF reported a 39.4% reduction in cough frequency after 14 days of twice daily treatment with low-dose morphine compared with placebo. Common side effects reported in these studies included nausea, constipation, and drowsiness [48, 57, 63]. The major drawback of the clinical use of morphine is the risk of physical dependence [48]. Morphine for pain management has contributed to a worldwide epidemic of opioid abuse, where prescriptions for MOR agonists often serve as a pathway to illicit drug use [39]. Other opioid receptor agonists have shown anti-tussive effects. For example, although both classified as Schedule IV of the Controlled Substances Act in the United States, pentazocine [64] and butorphanol [65], administered intravenously, were effective in managing the adverse effect of acute cough during anesthesia induction [66, 67]. Butorphanol has been approved as an anti-tussive agent for use in veterinary medicine [68].

Although codeine, a pro-drug of morphine, is often regarded as the “gold standard” for the management of cough, evidence from several studies shows a lack of effective cough suppression compared with placebo [69–71]. In one study [72], only ~ 17% of patients with chronic cough responded to codeine as a treatment for cough suppression. Codeine is 3-methylmorphine and undergoes de-methylation in the liver with a genetically variable first-pass metabolism with poor therapeutic response in some patients, likely due to variable metabolism [73]. Like morphine, codeine is also associated with the risk of abuse and, as a result, was recently re-classified as a prescription-only medicine in the United Kingdom [74]. Therapies that target KORs have also been studied to manage pain, depression, and anxiety [35]. Mixed opioid agonists or peripherally restricted KOR agonists have been effective in blocking the rewarding effects of morphine; therefore, they have therapeutic potential in managing drug abuse [35]. When administered subcutaneously in dogs, the anti-tussive effect of butorphanol was 4 times more potent than morphine and 100 times more potent than codeine; when administered orally, it was approximately 15–20 times more active than either codeine or dextromethorphan [75].

Given the neuroanatomical location of the KORs and MORs and the reported clinical trial results, mixed opioids as a drug class may target both the central and the peripheral neural pathways and could address a gap in the management of chronic cough. The mechanistic potential for synergistic pharmacological action in KORs and MORs, both centrally and peripherally in lung tissue, is promising for achieving clinically meaningful efficacy. This is in contrast with ongoing therapies aimed at addressing cough that primarily target a single opioid receptor, such as morphine, or those that target other receptors, such as P2X3 found on peripheral nerve endings [76]. Specifically, a therapy that targets both KORs and MORs could lead to synergistic anti-tussive effects without the potential for abuse. Opioid agonist–antagonist drugs that exhibit dual pharmacological actions by acting as agonists at KORs and antagonists at MORs were developed in a deliberate effort to create effective analgesic agents with less potential for abuse [77]. In support of this pharmacological approach, the parenterally administered opioid agonist–antagonist nalbuphine has demonstrated in a clinical study preventative anti-tussive effects for the management of acute cough during administration of sufentanil-induced anesthesia [78]. Furthermore, pentazocine and butorphanol have been effective as pre-treatments for fentanyl-induced cough in the perioperative setting [66, 79].

## A Novel Therapeutic Agent for Chronic Cough

The agonist–antagonist opioid moiety nalbuphine provides an additional synergistic possibility by acting simultaneously at the KORs and MORs. In its approved parenteral formulation, nalbuphine is not considered a controlled substance in the United States under the Controlled Substances Act [80]. In addition, at the international level, nalbuphine is not included in the List of Narcotic Drugs Under International Control [81]. Nalbuphine, approved in the parenteral formulation for the treatment of severe pain [82], is currently being investigated as an oral formulation for the management of chronic cough [83]. In a recent phase 2 clinical study of patients with chronic IPF-related cough, the use of nalbuphine extended-release (ER) tablets demonstrated a significant reduction in the frequency of IPF-associated cough. This short-term crossover trial consisted of two 22-day treatment periods, with participants with definite or probable IPF [83]. Participants were randomly assigned 1:1 to receive nalbuphine ER (27 mg once daily titrated to 162 mg twice daily) during period 1 followed by a crossover to placebo during period 2, or placebo during period 1 followed by nalbuphine ER during period 2. After the two 22-day treatment periods, the use of nalbuphine ER had significantly reduced the frequency of daytime cough, assessed objectively, by 75.1%, compared with 22.6% with placebo (*p* < 0.001) [83]. Furthermore, a 76.1% reduction (95% CI 83.1–69.1) was observed in the 24-h objective cough frequency with nalbuphine ER, compared with a 25.3% decrease (95% CI 43.9–6.7) with placebo [83]. Nalbuphine ER may have an advantage because it was designed to act synergistically both centrally in the brain and the brainstem and peripherally in the lungs to provide an anti-tussive effect independent of peripheral cough stimuli. A centrally acting agent may be effective across a broader spectrum of chronic cough phenotypes because the response is not dependent on a specific trigger.

## Conclusions

The substantial unmet need for an effective and well-tolerated treatment for chronic cough is challenging. The neurophysiological concept of sensitization, an important phenomenon in the field of pain, may be both an explanation for the development of chronic cough and point to opioid receptor-targeted therapy as an intervention. Opioid receptors have shown unique properties by which cough can be modulated. KORs and MORs are important therapeutic targets for cough suppression in CHS. Dual-effect opioids that have KOR agonism and MOR antagonism have unique properties across chronic sensory disorders and, thus, may reduce cough, with low sedation and low potential for drug abuse. Additional studies are necessary to elucidate the role of opioid receptors in the central and peripheral pathways of cough hypersensitivity and the role of mixed-effect opioids in managing chronic cough.

## Data Availability

No datasets were generated or analyzed during the current study.
